# Development of a predictive score for potentially avoidable hospital readmissions for general internal medicine patients

**DOI:** 10.1371/journal.pone.0219348

**Published:** 2019-07-15

**Authors:** Anne-Laure Blanc, Thierry Fumeaux, Jérôme Stirnemann, Elise Dupuis Lozeron, Aimad Ourhamoune, Jules Desmeules, Pierre Chopard, Arnaud Perrier, Nicolas Schaad, Pascal Bonnabry

**Affiliations:** 1 Pharmacy, Geneva University Hospitals, Geneva, Switzerland; 2 Pharmacie Interhospitalière de la Côte, Morges, Switzerland; 3 School of Pharmaceutical Sciences, University of Geneva, University of Lausanne, Geneva, Switzerland; 4 Groupement hospitalier de l’ouest lémanique (GHOL), Nyon, Switzerland; 5 Department of General Internal Medicine, Geneva University Hospitals, Geneva, Switzerland; 6 Division of Clinical Epidemiology, Geneva University Hospitals, Geneva, Switzerland; 7 Division of Quality of Care, Medical and Quality Directorate, Geneva University Hospitals, Geneva, Switzerland; 8 Clinical Pharmacology and Toxicology, Geneva University Hospitals, Geneva, Switzerland; University of Florence, ITALY

## Abstract

**Background:**

Identifying patients at high risk of hospital preventable readmission is an essential step towards selecting those who might benefit from specific transitional interventions.

**Objective:**

Derive and validate a predictive risk score for potentially avoidable readmission (PAR) based on analysis of readmissions, with a focus on medication.

**Design/Setting/Participants:**

Retrospective analysis of all hospital admissions to internal medicine wards between 2011 and 2014. Comparison between patients readmitted within 30 days and non-readmitted patients, as identified using a specially designed algorithm. Univariate and multivariate regression analyses of demographic data, clinical diagnoses, laboratory results, and the medication data of patients admitted during the first period (2011–2013), to identify factors associated with PAR. Using these, derive a predictive score with a regression coefficient-based scoring method. Subsequently, validate this score with a second cohort of patients admitted in 2013–2014. Variables were identified at hospital discharge.

**Results:**

The derivation cohort included 7,317 hospital stays. Multivariate logistic regressions found significant associations with PAR for: [adjusted OR (95% CI)] hospital length of stay > 4 days [1.3 (1.1–1.7)], admission in previous 6 months [2.3 (1.9–2.8)], heart failure [1.3 (1.0–1.7)], chronic ischemic heart disease [1.7 (1.2–2.3)], diabetes with organ damage [2.2 (1.3–3.8)], cancer [1.4 (1.0–1.9)], metastatic carcinoma [1.9 (1.3–3.0)], anemia [1.2 (1.0–1.5)], hypertension [1.3 (1.1–1.7)], arrhythmia [1.3 (1.0–1.6)], hyperkalemia [1.4 (1.0–1.7)], opioid drug prescription [1.3 (1.1–1.6)], and acute myocardial infarction [0.6 (0.4–0.9)].

The PAR-Risk Score, derived from these results, demonstrated fair discriminatory and calibration power (C-statistic = 0.699; Brier Score = 0.069). The results for the validation cohort’s operating characteristics were similar (C-statistic = 0.687; Brier Score = 0.064).

**Conclusion:**

This study identified routinely-available factors that were significantly associated with PAR. A predictive score was derived and internally validated.

## Background

In the USA, as many as one in five patients risks being readmitted within 30 days of hospital discharge, with an annual estimated extra cost to the healthcare system of USD 26 billion [1, 2]. A significant proportion of these readmissions could be prevented (5%–79%) [3] and may be the consequence of suboptimal continuity of care [4]. Similar problems are observed in the Swiss healthcare system, dealing with difficulties of care coordination and suboptimal continuity of care when hospitalization occurs. Several interventions can be implemented to prevent hospital readmissions, the majority of them dealing with care coordination [5]. Reducing readmission rates by targeting care coordination interventions towards high-risk patients seems to be an efficient strategy, particularly when resources are limited [6, 7]. High-risk patients should, therefore, be identified early during their hospital stay in order to benefit most from specific interventions.

Recently, various scoring methods have been shown to predict 30-day readmission in general internal medicine patients [8–15]. However, only one of them focused on potentially avoidable hospital readmission (PAR) [8]. Today, PAR is considered a good indicator on which to work to reduce overall readmissions that may indeed be avoidable [16]. Of course, many unplanned readmissions are unavoidable, such as those for new medical conditions unrelated to any previous diagnosis or readmissions for transplantation or delivery; these do not need to be targeted [17].

In the USA, the Hospital Readmission Reduction Program recently introduced financial incentives for reducing readmissions associated with particular diagnoses; it targets higher than expected 30-day readmission rates for selected medical conditions [18]. A similar system was introduced in Switzerland’s hospital financial system in 2012 (Swiss DRG rules). Consequently, hospital readmission for the same major diagnosis category within 18 days of discharge now prevents any reimbursement to the hospital. Thus, limiting hospital readmissions has become a significant challenge for hospitals in Switzerland, as it has in most developed-country healthcare systems, whatever their financing model.

Among other factors, adverse drugs events can contribute to hospital readmission [19, 20], and several drug classes have been associated with this [21]. Significantly, not all the published available scores specifically include medication profiles and drug exposure, thus creating important uncertainties regarding the magnitude of the medication’s involvement in PAR. We therefore undertook this study in order to derive and validate a predictive model of PAR that includes medication profiles and the identification of the specific characteristics of patients readmitted to the general internal medicine wards of two Swiss hospitals.

## Method

### Study design and population

This retrospective, observational, two-center study included all the patients admitted to the general internal medicine wards of the Geneva University Hospitals (HUG) and the regional hospital in Nyon (Groupement Hospitalier de l’Ouest Lémanique, GHOL), who were discharged alive, and were not transferred to any other acute-care hospital. The study covered two consecutive periods: the derivation cohort was discharged between December 1, 2011, and November 30, 2013; the validation cohort was discharged between December 1, 2013, and November 30, 2014.

PAR in both cohorts was identified using the SQLape algorithm, a system used in Swiss hospitals for benchmarking and national quality of care surveys. This system is based on administrative data, medical procedures, and diagnoses as coded in the International Classification of Disease, 10^th^ edition (ICD-10). It can identify unplanned readmissions to the same hospital (to any department), related to the initial diagnosis, and occurring within 30 days of hospital discharge [16, 22]. The algorithm does not identify as PAR any planned readmissions, readmissions for transplantation, labor and delivery, chemotherapy or radiotherapy, follow-up or rehabilitation treatments, readmissions for medical conditions involving damage to a new organ system that were not present during the index admission, and readmissions for trauma or severe chronic diseases (multiple sclerosis, liver cirrhosis, urinary calculus, etc.) [23]. The SQLape screening algorithm’s specificity and sensitivity are 96% in comparison to manual analysis of medical records [22].

### Data collection

Administrative data were retrospectively extracted from the two institutions’ administrative databases (DPI, an in-house electronic patient record, for the HUG, and Opale, OrdiConseil, Geneva, for the GHOL). Data included date of birth, hospital length of stay (LOS), hospital admission in the previous 6 months, and elective versus urgent hospital admission. Clinical data extracted from electronic patient records included medical diagnoses, medication prescriptions, and the last available laboratory results. Primary and secondary diagnoses in the medical coding were used to identify patients’ comorbidities (variable definitions are provided in S1 Table) and to calculate the Charlson’s Comorbidity Index (as it is possible to assess this comorbidity index from administrative databases) [24–26]. Medications prescribed during the last day in hospital were retrieved from electronic prescription software (DPI-Presco, for the HUG; Predimed or Cerner Soarian Clinical, for the GHOL). All the collected data were anonymized.

### Statistical analysis

Descriptive statistics (means, proportions, standard deviations) were used for all variables. The baseline characteristics of PAR patients and non-readmitted patients were compared using the Chi-square test for association. Based on the parameters identified using this first analysis, the list of predictive factors to be studied was restricted to those with clinical relevance and non-redundant information (i.e., the Charlson Comorbidity Index score and hospital origin were not analyzed for redundant information about comorbidities *per se* and non-relevant data, respectively). Other previously published predictive factors were also considered and included in the list. The variables of interest were then included in a multivariate logistic regression model and selected using a stepwise (backwards) algorithm with a *p*-value < 0.2 as the stopping criteria.

C-statistics, the Brier Score, and the Hosmer-Lemeshow chi-square test were used to assess the discrimination and calibration of the final multivariate model for both the derivation and validation cohorts. Internal validation based on a bootstrap method was used on the derivation cohort to correct optimism of the observed discrimination and calibration performance of the model.

Then the final reduced model was used to develop a predictive score, based on a regression-coefficient model. The scoring system was derived by multiplying each beta coefficient by ten and rounding to the nearest integer. Next, each patient’s total score was obtained by adding all the integers from the applicable variables C-statistics of the predictive score were computed for the derivation and validation cohorts, as well as Brier Score and Hosmer-Lemeshow test using the predicted probabilities from a univariate logistic regression model with the score as unique covariate and the readmission status as outcome.

All statistical analyses were performed using R software (R Core Team (2016); R: A language and environment for statistical computing. R Foundation for Statistical Computing, Vienna, Austria. URL https://www.R-project.org/).

### Ethics committee approval

The study protocol was approved by the Ethics Committee on Health Research *(CEREH)*, for the HUG, and by the Human Research Ethics Committee of the Canton Vaud, for the GHOL.

## Results

**Fig 1:**
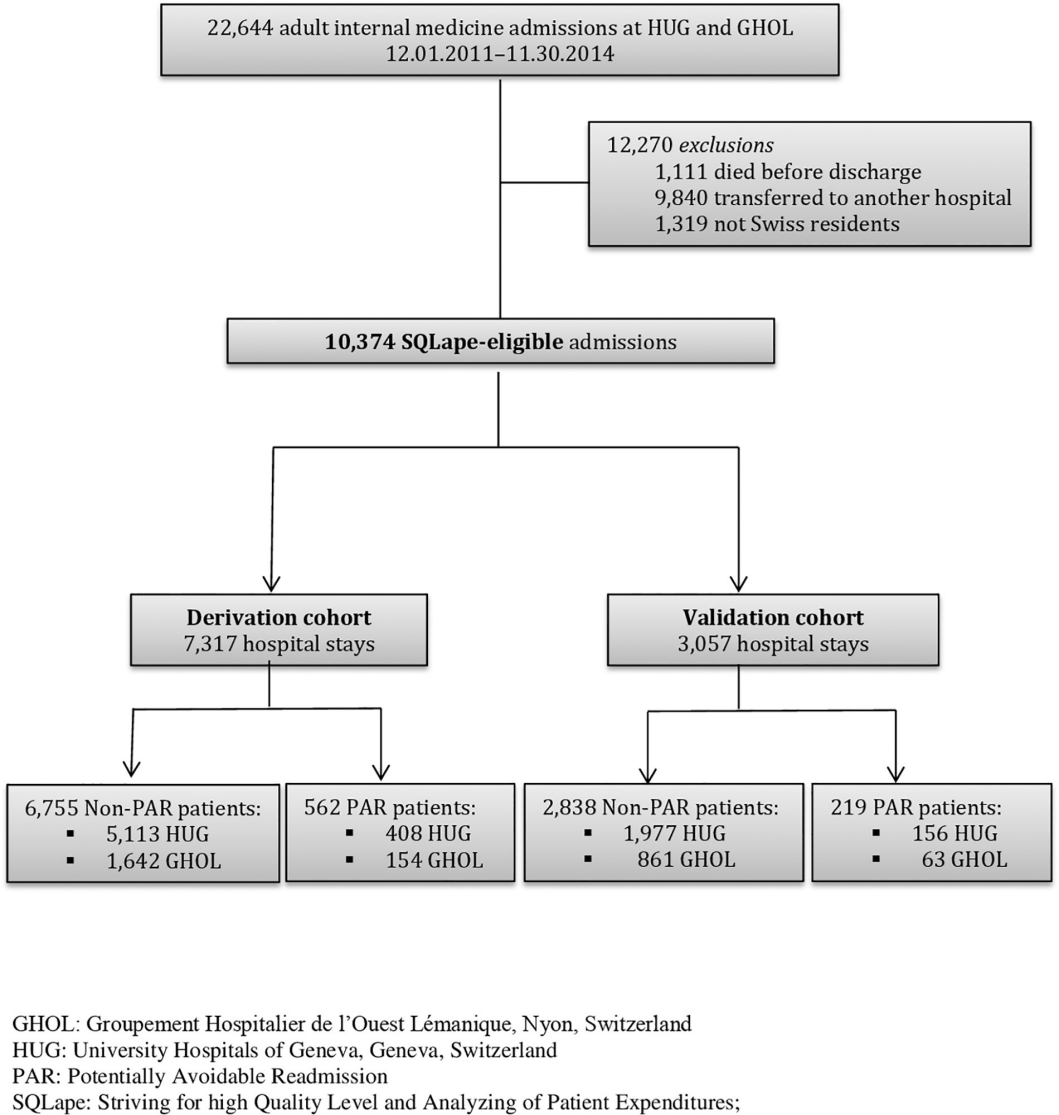
Study flowchart of all analyzed patients.

The derivation and validation cohorts included a total of 10,374 inpatient hospital stays. Of these, 781 (7.5%) were identified as followed by a PAR (Fig 1).

The derivation cohort included patient data from 7,317 hospital stays, of which 562 were followed by a PAR (7.6%). The derivation cohort’s baseline clinical and demographic variables are presented in Table 1. The mean age of derivation cohort patients was 66.5 years old (SD 18.5); 55% were male; 43% were prescribed 10 or more different medications.

**Table 1:** Baseline characteristics of the derivation cohort.

Baseline Characteristics at hospital discharge	Derivation cohort (n = 7317)	
	No.	%
University Hospitals of Geneva	5521	(75.5)
Groupement Hospitalier de l’Ouest Lémanique	1796	(24.6)
**Age**		
≤ 65 years	2866	(39.2)
66–75 years	1555	(21.3)
≥ 76 years	2896	(39.6)
**Male sex**	3993	(54.6)
**Charlson Comorbidity Index**		
≤ 1	4094	(56.0)
2–4	2561	(35.0)
> 4	662	(9.0)
**Length of hospital stay**		
≤ 4 days	2259	(30.9)
> 4 days	5058	(69.1)
**Type of admission**		
Unplanned/emergency	6331	(86.5)
Planned	585	(8.0)
Transfer/others	401	(5.5)
**Admission in previous 6 months**	2041	(27.9)
**Comorbidity**:		
Acute myocardial infarction	1048	(14.3)
Acute respiratory disease	1260	(17.2)
AIDS	25	(0.3)
Anemia	2138	(29.2)
Arrhythmia	1342	(18.3)
Cancer	762	(10.4)
Metastatic carcinoma	280	(3.8)
Cerebrovascular disease	268	(3.7)
COPD/asthma	1043	14.3
Chronic ischemic heart disease	497	(6.8)
Cognitive troubles/dementia	201	(2.8)
Connective tissue disease	64	(0.9)
Diabetes with organ damage	152	(2.1)
Gastrointestinal ulcer	100	(1.4)
Hepatic cirrhosis	276	(3.8)
Heart failure	1314	(18.0)
Hypertension	1723	(23.6)
Infectious disease (except pneumonia and sepsis)	1655	(22.6)
Intoxication or adverse drug reactions	918	(12.6)
Mental and behavioral disorders due to alcohol	639	(8.7)
Paraplegia/hemiplegia	84	(1.2)
Peripheral vascular disease	186	(2.5)
Pneumonia	1353	(18.5)
Renal failure	1678	(22.9)
Sepsis	542	(7.4)
**Number of medications**		
< 5	1671	(22.8)
6 to 10	2469	(33.7)
> 10	3177	(43.4)
**Main drugs prescribed**		
ACE inh./ angiotensin II antag.	2773	(37.9)
Antiplatelet drugs	2575	(35.2)
Anticoagulants	1170	(16.0)
Antipsychotics	571	(7.8)
Benzodiazepines	3271	(44.7)
Beta blockers	2322	(31.7)
Calcium-channel blockers	1367	(18.7)
Digoxin	201	(2.8)
Diuretics	2435	(33.3)
Hypoglycemic drugs (insulin/sulfonylurea/glinide)	1061	(14.5)
Non-secretagogue antidiabetics	739	(10.1)
NSAIDs	656	(9.0)
Opioids	1795	(24.5)
Systemic anti-infectious drugs	3268	(44.7)
**Laboratory analysis**		
Hyperkalemia (K > 5.5 mmol/L)	685	(9.4)
Hypokalemia (K < 3.5 mmol/L)	2839	(38.8)
Hypernatremia (Na > 145 mmol/L)	348	(4.7)
Hyponatremia (Na < 135 mmol/L)	2385	(32.6)
Liver dysfunction (ASAT/ALAT > 175; or total bilirubin > 40; or AP > 360; or GGT > 90)	1631	(22.3)

AIDS: acquired immune deficiency syndrome; COPD: chronic obstructive pulmonary disease; ACE inhibitors: angiotensin-converting-enzyme inhibitors; NSAID: nonsteroidal anti-inflammatory drugs; ASAT: aspartate amino transferase; ALAT: alanine amino transferase; GGT: gamma-glutamyl transferase; AP: alkaline phosphatase;

NA = non-applicable, descriptive data not included in univariate analysis, PAR = potentially avoidable readmission

Univariate analysis gave a list of possible predictive factors, completed using the literature, and making a total of 48 potential items. After a stepwise multivariate analysis, 13 items were shown to be independently and significantly associated with an increased risk of PAR. The univariate and multivariate analyses are presented in Table 2 (details of the multivariate analyses are presented in S2 Table).

**Table 2:** Univariate and multivariate analyses of the derivation cohort.

					Univariate analysis of the derivation cohort		Multivariate analysis of the derivation cohort	
Baseline Characteristics	Non-PAR patients (n = 6755)		PAR patients (n = 562)		Unadjusted OR (95% CI)		Adjusted OR (95% CI)	
	No.	%	No.	%				
**Age**								
≤ 65 years	2683	(39.7)	183	(32.6)	**1.0**			
66–75 years	1404	(20.8)	151	(26.9)	**1.58**	**(1.26–1.97)**	1.22	(0.95–1.56)
≥ 76 years	2668	(39.5)	228	(40.6)	**1.25**	**(1.02–1.53)**	0.92	(0.72–1.18)
**M–ale sex**	3683	(54.5)	310	(55.2)	1.03	(0.86–1.22)	0.96	(0.8–1.16)
**Length of hospital stay**								
≤ 4 days	2137	(31.6)	122	(21.7)	**1.67**	**(1.36–2.06)**	**1.31**	**(1.04–1.66)**
> 4 days	4618	(68.4)	440	(78.3)				
**Admission in previous 6 months**	1763	(26.1)	278	(49.5)	**2.77**	**(2.33–3.30)**	**2.30**	**(1.91–2.77)**
**Comorbidity**:								
Acute myocardial infarction	964	(14.3)	84	(15.0)	1.06	(0.82–1.34)	**0.64**	**(0.44–0.91)**
Acute respiratory disease	1140	(16.9)	120	(21.4)	**1.34**	**(1.08–1.65)**	1.05	(0.83–1.31)
AIDS	23	(0.3)	2	(0.4)	1.04	(0.17–3.54)	1.31	(0.20–4.63)
Anemia	1913	(28.3)	225	(40.0)	**1.69**	**(1.42–2.02)**	**1.25**	**(1.03–1.53)**
Arrhythmia	1197	(17.7)	145	(25.8)	**1.62**	**(1.32–1.96)**	**1.30**	**(1.02–1.65)**
Cancer	658	(9.7)	104	(18.5)	**2.10**	**(1.67–2.63)**	**1.41**	**(1.04–1.89)**
Metastatic carcinoma	230	(3.4)	50	(8.9)	**2.77**	**(1.99–3.78)**	**1.98**	**(1.32–2.96)**
Cerebrovascular disease	250	(3.7)	18	(3.2)	0.86	(0.51–1.36)	0.98	(0.55–1.63)
Chronic ischemic heart disease	430	(6.4)	67	(11.9)	**1.99**	**(1.50–2.60)**	**1.70**	**(1.24–2.31)**
Cognitive troubles/dementia	188	(2.8)	13	(2.3)	0.83	(0.45–1.40)	0.81	(0.42–1.43)
Connective tissue disease	57	(0.8)	7	(1.3)	1.48	(0.61–3.05)	1.54	(0.62–3.27)
COPD/asthma	956	14.2	87	15.5	1.11	(0.87–1.40)	1.27	(0.97–1.66)
Diabetes with organ damage	124	(1.8)	28	(5.0)	**2.80**	**(1.81–4.20)**	**2.25**	**(1.30–3.81)**
Gastrointestinal ulcer	89	(1.3)	11	(2.0)	1.50	(0.75–2.69)	1.35	(0.66–2.54)
Heart failure	1,155	(17.1)	159	(28.3)	**1.91**	**(1.57–2.32)**	**1.31**	**(1.01–1.70)**
Hepatic cirrhosis	245	(3.6)	31	(5.5)	**1.55**	**(1.04–2.24)**	1.32	(0.80–2.15)
Hypertension	1,551	(23.0)	172	(30.6)	**1.48**	**(1.22–1.78)**	**1.34**	**(1.06–1.68)**
Infectious disease (except pneumonia and sepsis)	1538	(22.8)	117	(20.8)	0.89	(0.72–1.10)	0.83	(0.65–1.04)
Intoxication or adverse drug reactions	827	(12.2)	91	(16.2)	**1.39**	**(1.09–1.75)**	1.18	(0.91–1.50)
Mental and behavioral disorders due to alcohol	590	8.7	49	(8.7)	0.99	(0.73–1.34)	0.99	(0.66–1.44)
Paraplegia/hemiplegia	77	(1.1)	7	(1.3)	1.09	(0.46–2.22)	1.54	(0.61–3.36)
Peripheral vascular disease	162	(2.4)	24	(4.3)	**1.81**	**(1.14–2.76)**	1.37	(0.84–2.16)
Pneumonia	1237	(18.3)	116	(20.6)	1.16	(0.93–1.43)	1.22	(0.95–1.56)
Renal failure	1505	(22.3)	173	(30.8)	**1.55**	**(1.28–1.87)**	1.11	(0.89–1.39)
Sepsis	504	(7.5)	38	(6.8)	0.90	(0.63–1.25)	0.74	(0.50–1.07)
**Number of medications**								
< 5	1569	(23.2)	102	(18.2)	1.00			
6–10	2302	(34.1)	167	(29.7)	1.12	(0.87–1.44)	0.90	(0.69–1.18)
> 10	2884	(42.7)	293	(52.1)	**1.56**	**(1.24–1.98)**	0.97	(0.73–1.31)
**Main drugs prescribed**								
ACE inh./ angiotensin II antag.	2540	(37.6)	233	(41.5)	1.18	(0.99–1.40	0.93	(0.75–1.15)
Antiplatelet drugs	2356	(34.9)	219	(39.0)	1.19	(0.99–1.42)	0.89	(0.71–1.11)
Anticoagulants	1066	(15.8)	104	(18.5)	1.21	(0.97–1.51)	0.92	(0.71–1.19)
Antipsychotics	534	(7.9)	37	(6.6)	0.82	(0.57–1.14)	0.91	(0.62–1.30)
Benzodiazepines	3022	(44.7)	249	(44.3)	0.98	(0.83–1.17)	0.84	(0.70–1.02)
Beta blockers	2099	(31.1)	223	(39.7)	**1.46**	**(1.22–1.74)**	1.01	(0.81–1.27)
Calcium-channel blockers	1233	(18.3)	134	(23.8)	**1.40**	**(1.14–1.71)**	1.04	(0.82–1.30)
Digoxin	179	(2.7)	22	(3.9)	1.50	(0.93–2.30)	1.26	(0.76–2.00)
Diuretics	2183	(32.3)	252	(44.8)	**1.70**	**(1.43–2.03)**	1.13	(0.89–1.42)
Hypoglycemic drugs (insulin/sulfonylurea/glinide)	960	(14.2)	101	(18.0)	**1.32**	**(1.05–1.65)**	1.13	(0.83–1.54)
Non-secretagogue antidiabetics	690	(10.2)	49	(8.7)	0.84	(0.61–1.13)	0.87	(0.61–1.23)
NSAIDs	616	(9.1)	40	(7.1)	0.76	(0.54–1.05)	1.10	(0.75–1.55)
Opioids	1616	(23.9)	179	(31.9)	**1.49**	**(1.23–1.79)**	**1.33**	**(1.07–1.63)**
Systemic anti-infectious drugs	3020	(44.7)	248	(44.1)	0.98	(0.82–1.16)	1.00	(0.81–1.25)
**Laboratory analysis**								
Hyperkalemia (K > 5.5 mmol/L)	595	(8.8)	90	(16.0)	**1.97**	**(1.54–2.50)**	**1.35**	**(1.02–1.77)**
Hypokalemia (K < 3.5 mmol/L)	2623	(38.8)	216	(38.4)	0.98	(0.82–1.17)	0.88	(0.72–1.07)
Hypernatremia (Na > 145 mmol/L)	318	(4.7)	30	(5.3)	1.14	(0.76–1.65)	0.98	(0.63–1.45)
Hyponatremia (Na < 135 mmol/L)	2176	(32.2)	209	(37.1)	**1.25**	**(1.04–1.49)**	1.05	(0.86–1.27)
Liver dysfunction (ASAT/ALAT > 175; or total bilirubin > 40; or AP > 360; or GGT > 90)	1491	(22.1)	140	(24.9)	1.17	(0.95–1.43)	0.99	(0.79–1.25)

AIDS: acquired immune deficiency syndrome; COPD: chronic obstructive pulmonary disease; ACE inhibitors: angiotensin-converting-enzyme inhibitors; NSAID: non-steroidal anti-inflammatory drugs; ASAT: aspartate aminotransferase; ALAT: alanine aminotransferase; GGT: gamma-glutamyl transferase; AP: alkaline phosphatase;

NA = non-applicable, descriptive data not included in univariate analysis, PAR = potentially avoidable readmission

**In bold**: significant results (*p* < 0.05).

Based on these results, we created a 12-item prediction model. The PAR-Risk Score (**P**otentially **A**voidable **R**eadmissions **R**isk **S**core) includes the following variables: hospital LOS > 4 days, admission in previous 6 months, anemia, hypertension, hyperkalemia, opioid prescription during hospital stay, diagnosis of or a comorbidity with heart failure, acute myocardial infarction, chronic ischemic heart disease, diabetes with organ damage, cancer, and metastatic carcinoma (Table 3; S2 Table describes beta coefficients and odds ratios for all the included predictors). A specific automatic calculator that can estimate patients’ specific risks of readmission based on their PAR-Risk Score was developed on an Excel spreadsheet (S3 Table).

**Table 3:** Potentially avoidable readmission risk score (PAR-Risk score).

Characteristics	Points
***Administrative characteristics***	
Admission in previous 6 months	8
Hospital length of stay > 4 days	3
***Comorbidities***	
Anemia	2
Heart failure	4
Hypertension	3
Acute myocardial infarction	- 4
Chronic ischemic heart disease	5
Diabetes with organ damage	9
Cancer	4
Metastatic carcinoma	6
***Medications***	
Opioids	3
***Lab results***	
Hyperkalemia (> 5.5 mmol/L)	4
**Total maximum**	**47**

The PAR-Risk Score makes it possible to divide the risk of PAR into three tertiles, namely low, intermediate, and high risk.

The PAR-Risk Score was then applied to the validation cohort (validation cohort baseline clinical and demographic characteristics are presented in S4 Table). Observed and predicted 30-day risks of PAR for both cohorts are described in Table 4.

**Table 4:** Observed versus predicted risk of 30-day potentially avoidable readmissions.

Points	Risk category	Patient distribution No. (%)		Observed risk %	Predicted risk %
**Derivation cohort**					
< 3	Low	1,182	(16.2%)	2.6	3.1
3–10	Medium	3,420	(46.7%)	5.2	5.0
> 10	High	2,715	(37.1%)	12.9	13.1
**Validation cohort**					
< 3	Low	530	(17.2%)	2.4	3.5
3–10	Medium	1,370	(44.8%)	5.7	5.7
> 10	High	1,156	(37.8%)	13.1	13.9

The final multivariate model’s discrimination performance was acceptable with a C-statistic of 0.699 (95% CI: 0.677–0.721) and of 0.674 after correction for optimism by bootstrap in the derivation cohort; the C-statistic showed a similar value in the validation cohort 0.688 (95%CI: 0.655–0.722). The Brier Score was equal to 0.068 (0.069 after correction for optimism) and 0.066 in the derivation and validation cohorts, respectively. However, the Hosmer-Lemeshow test was significant, with a p-value of 0.023 and 0.002 in the derivation and validation cohorts, respectively.

The PAR-Risk Score had a C-statistic of 0.699 (95% CI: 0.676–0.721) in the derivation cohort and 0.687 (95% CI: 0.654–0.721) in the validation cohort. The Brier Score of the PAR-Risk Score was equal to 0.068 in the derivation cohort and to 0.065 in the validation cohort. The Hosmer-Lemeshow test was significant in both cohorts (p = 0.004 and p = 0.003, respectively). The predicted number of potentially avoidable hospital readmissions by decile are presented in S5 Table, for both the derivation and validation cohorts.

## Discussion

The present study, based on 10,374 hospital admissions to two general internal medicine wards, derived and internally validated the PAR-Risk Score to identify patients at a high risk of PAR within 30 days of discharge. The PAR-Risk Score showed an acceptable ability to discriminate patients into low, medium, and high-risk categories, with C-statistic values comparable to those reported in a recent systematic review [17]. As a next step, therefore, we think that the PAR-Risk Score should be validated externally in order to confirm the interest of its use in detecting patients at a high risk of PAR.

The focus on PAR was deliberate: we believe that targeting this type of readmission is an efficient way to decrease overall readmission rates. All the currently proposed interventions for decreasing readmission rates are resource intensive (in time and personnel) and therefore cannot reasonably be applied to all patients [27]. Non-preventable readmissions represent a significant proportion of total readmissions, as previously reported in the literature [3]. It is impossible, by definition, to reduce non-preventable readmission rates; thus, special attention should be paid to readmissions that can indeed be avoided. A mean rate of PAR of around 7.5% was observed in both the hospitals involved in this study, very similar to previously published ranges, particularly a recent publication by Donzé *et al*., suggesting that our results are probably relevant [3, 8].

The PAR-Risk Score is based on easily obtainable data which can be collected soon after hospital admission and whose values can evolve during the stay. Considering the variables included in the PAR-Risk Score, its automatic integration into electronic patient records might be an interesting future strategy to obtain prospective data and evaluate specific interventions that decrease hospital readmissions.

As per our definition, LOS is the only variable that is only collected after day four. Other PAR-Risk Score parameters reflect health resource utilization, administrative information, and comorbidities. In the literature, all these parameters have already been associated with an increased risk of hospital readmission. Many of them have already been included in other prediction scores, such as the LACE index [15] and the more recent HOSPITAL score [8]. In the PAR-Risk Score, hospital stays longer than 4 days are associated with PAR readmissions. Indeed, hospital LOS has previously been associated with hospital readmissions [17, 28], but with variable cut-off values, ranging from 2 or 3 days [28–30] to 7 days [13, 31]. The 4-day cut-off was mediated from data identified in various previously published scores, but especially the HOSPITAL score as it focused on hospital potentially preventable readmissions. This cut-off determines a short vs. long LOS that can be correlated with the severity of the disease. Admission in the previous 6 months has also been previously identified as a strong predictor of readmission [10, 17], and it is included in the HOSPITAL score and the LACE index [8, 15, 17].

Similarly, our predictive model confirms the importance of existing comorbidities; they are risk factors strongly associated with both hospital readmissions and PAR [10, 14, 15, 17, 32–34]. Heart failure has been associated with an increased risk of readmission [10, 14, 33, 35, 36] and is one of the conditions, together with myocardial infarction, that has been the focus of significant financial penalties associated with high readmission rates [35, 37]. Surprisingly, however, the PAR-Risk Score for acute myocardial infarction showed this condition to be associated with a lower risk of PAR, which was not the case for chronic ischemic heart disease. This may be partly explained by the fact that the usual clinical management and follow-up of acute myocardial infarction are probably more concordant with guidelines than are those for chronic ischemic disease, and by the fact that recurrent symptomatology is probably more frequent in the latter [38], [39]. Another explanation could be related to the high number of planned readmissions in post-acute myocardial infarction patients (e.g., planned coronary artery bypass grafts), which lowers their risk of PAR. Oncological comorbidities, anemia, hypertension, and diabetes have also been frequently associated with an increased risk of hospital readmission and sometimes with PAR [8, 10, 13, 14, 33, 40].

Medication issues, such as drug-related problems and inappropriate medication, have also been associated with hospital readmission [21, 41–43], suggesting that these variables should be included in prediction models [21]. For this reason, we specifically addressed these issues in our analyses. The results of our multivariate analyses showed that the only drug class associated with a significant increase in the risk of PAR were opioid drugs. There is a risk of adverse outcomes with these analgesics, especially in elderly patients [44, 45], [46], including accidental overdose, over-sedation, or respiratory depression [45]. This has led to the inclusion of opioid drugs in predictive tools for the risk of death and hospital admission among elderly people [10, 47].

Although it has never been described as a risk factor in the past, our study also associated hyperkalemia with PAR. This association may reflect the use of angiotensin-converting enzyme inhibitors/angiotensin II antagonists (ACEI/ARA) and/or spironolactone, or perhaps the presence of comorbidities already included in our analysis, such as heart or renal failure.

The PAR-Risk Score has a number of strengths in comparison to the many scores and prediction models published in recent years, the majority of which were developed to identify patients at a high risk of 30-day readmission [17]. The PAR-Risk Score was designed to detect any increased risk of PAR, an outcome that only the HOSPITAL score considered previously [8, 22].

The PAR-Risk Score is also one of the few scores developed from the analysis of a population of patients outside the USA, probably making it applicable in countries with hospital systems comparable to Switzerland’s. The only other score validated on the Swiss population is the HOSPITAL score [48]. Moreover, the PAR-Risk score’s development was based on the analysis of two different types of hospitals—one tertiary university teaching hospital and one regional hospital.

The HOSPITAL score cannot be used until the end of the hospital stay because its variables include “low hemoglobin level at discharge”, “discharge from an oncology service”, and “low sodium level at discharge”. These variables cannot be obtained before the last day. Furthermore, the number of hospital admissions during the previous year is quite complicated to obtain from Swiss hospitals as it is not a routinely available variable. In light of this aspect, our variables (anemia, admission in previous 6 months, …etc.) are easier to obtain throughout hospital stays. The PAR-Risk score can therefore be applied earlier in the hospital stay and allow transitional support measures to be implemented in time. The PAR-Risk Score is thus similar to the HOSPITAL score, with the exception that medication is included in the univariate and multivariate analyses.

Considering their performance, both score are similar when regarding discriminatory power (C-test: 0.70 for PAR-Risk Score, 0.69 and 0.70 for HOSPITAL score and simplified-HOSPITAL respectively [8, 48] and for the overall accuracy (Brier Score: 0.068 for PAR-Risk Score, 0.10 and 0.10 for HOSPITAL and simplified-HOSPITAL score respectively [8]. However, considering calibration with the Hosmer-Lemeshow test, it is better with the HOSPITAL and simplified-HOSPITAL score than with the PAR-Risk score (Hosmer-Lemeshow test: 0.004 for the PAR-Risk score, 0.28 and 0.40 for the HOSPITAL and simplified HOSPITAL score respectively [8, 48]. As Brier Score depends on the prevalence of the outcome (here the proportion of readmission), we computed the Brier Score of the simplified HOSPITAL on our derivation cohort and it was equal to 0.067. Considering Hosmer-Lemeshow test, it was also significant for the HOSPITAL score on our derivation cohort (p < 0.001). So both score have similar performance on our sample.

Certain limitations to the present study’s results do need to be addressed. First, many data were extracted from administrative databases and thus highly dependent on the quality of documentation and medical coding. However, we are confident of their quality, as administrative coding processes are strictly and regularly monitored by Switzerland’s national authorities. Neither of the hospitals in our study has been reported for any deviations from the control procedures. Second, the identification of PAR was performed within the inherent limits of the SQLape algorithm. However, the system’s robustness and good operating characteristics (96% sensitivity and specificity) [22] make us very confident about the process of identifying PAR in our population. Third, one of SQLape’s limitations is that patients transferred to rehabilitation are not included in the analysis; indeed, such patients are usually polymorbid and polymedicated, clearly making them at risk of PAR. Further studies should thus focus on this population too. Another of SQLape’s limitations is that patients readmitted to different hospitals cannot be identified and included in the analysis. Fourth, our model did not include parameters such as functional status, social support, health literacy, socioeconomic conditions, medication regimen complexity, or reported medication adherence. These parameters are rarely reported and difficult to obtain in a retrospective analysis, but they merit attention in the future.

Fifth, hospital LOS, hyperkaliemia electrolyte disorder, and medication prescribed at discharge are the three variables included in our PAR-Risk score that cannot be obtained before the discharge day. However, most of the comorbidities included are detectable earlier in the hospital stay, except for any new medical conditions. Another variable available earlier during the stay is previous hospital admission. A trend can therefore be drawn from the earlier stages of the hospital stay and must be confirmed throughout it in order to implement specific transitional interventions for reducing numbers of hospital readmissions. This limitation is common to most readmission score, including the HOSPITAL score. Finally, we only performed an internal retrospective validation of our predictive score; external and prospective validation of the PAR-Risk Score will be required before it can be put to clinical use.

## Conclusion

In conclusion, based on over 10,300 hospital stays, we derived and internally validated the PAR-Risk Score to predict the risk of potentially avoidable readmission. Although the PAR-Risk Score does not have perfect discriminating power, partly due to methodological issues, it could nevertheless be proposed as a screening tool to identify high-risk patients [6, 7, 49–51]. Focusing on PAR to decrease the overall readmission rate seems to be a reasonable approach with which to limit the use of human and time resources in transitional care processes. Including medication variables in the PAR-Risk Score is in accordance with published data on the negative role of drug profiles on outcomes during the transition of care after hospital discharge [20, 52, 53]. The PAR-Risk Score may help to identify high-risk patients before discharge home, and this should help healthcare providers to target complex transitional interventions that improve the coordination of care with the overarching goal of decreasing readmission rates [6].

## Supporting information

S1 Table Definitions for variables of interest. Adapted from *Canadian Institute for Health Information*, *Hospital Standardized Mortality Ratio*: *Technical Notes*, *2011*. *S1 Table*: Variable of interest’s definition.
(DOCX)

S2 Table Multivariate analysis with all variables of interest. AIDS: acquired immune deficiency syndrome; COPD: chronic obstructive pulmonary disease; ACE inhibitors: angiotensin-converting-enzyme inhibitors; NSAID: nonsteroidal anti-inflammatory drugs; ASAT: aspartate aminotransferase; ALAT: alanine aminotransferase; GGT: gamma-glutamyl transferase; AP: alkaline phosphatase. In bold: significant result (*p* < 0.05). *S2 Table*: Multivariate analysis with all variables of interest.
(DOCX)

S3 Table Calculator of predicted potentially avoidable readmission risk. *S3 Table*: Calculator of predicted potentially avoidable readmission risk (+ *Excel* spreadsheet).
(DOCX)

S4 Table Validation cohort’s baseline characteristics. AIDS: acquired immune deficiency syndrome; COPD: chronic obstructive pulmonary disease; ACE inhibitors: angiotensin-converting-enzyme inhibitors; NSAID: nonsteroidal anti-inflammatory drugs; ASAT: aspartate aminotransferase; ALAT: alanine aminotransferase; GGT: gamma-glutamyl transferase; AP: alkaline phosphatase; PAR: potentially avoidable readmission. *S4 Table*: Baseline characteristics of the validation cohort.
(DOCX)

S5 Table Predicted number of potentially avoidable hospital readmissions by decile, in both derivation and validation cohorts. A) In derivation cohort (n = 7,317) B) In validation cohort (n = 3,057). *S5 Table*: Predicted number of potentially avoidable hospital readmissions by decile, in both derivation and validation cohorts.
(DOCX)
