# Supplementary material for: Development of a predictive score for potentially avoidable hospital readmissions for general internal medicine patients
Source: PLoS One. 2019 Jul 15;14(7):e0219348. doi: 10.1371/journal.pone.0219348 (PMC6629067; doi:10.1371/journal.pone.0219348)
Supplement: S1 Table — Adapted from Canadian Institute for Health Information, Hospital Standardized Mortality Ratio: Technical Notes, 2011. S1 Table: Variable of interest’s definition. (DOCX) [file pone.0219348.s001.docx]

**S Table 1: Definitions for variables of interest**

| **Variables of interest** | **Definition (ICD-10 codes)** | |
| --- | --- | --- |
| Acute myocardial infarction | I21*; I22*; I25.2* | |
| Acute respiratory disease | J9*; J8*; J6* | |
| AIDS | B20*; B21*; B22*; B24* | |
| Anemia | D5*; D6*; D7*; D46* or Hb < 90 g/L | |
| Arrhythmia | R00*; I44*; I47*; I48*; I49*; I45* | |
| COPD/asthma | J44*; J47*; J45*; J46*; J40*; J42*; J43*; J41* | |
| Cancer | C00*; C01*; C02*; C03*; C04*; C05*; C06*; C07*; C08*; C09*; C10*; C11*; C12*; C13*; C14*; C15*; C16*; C17*; C18*; C19*; C20*; C21*; C22*; C23*; C24*; C25*; C26*; C30*; C31*; C32*; C33*; C34*; C37*; C38*; C39*; C40*; C41*; C43*; C45*; C46*; C47*; C48*; C49*; C50*; C51*; C52*; C53*; C54*; C55*; C56*; C57*; C58*; C60*; C61*; C62*; C63*; C64*; C65*; C66*; C67*; C68*; C69*; C70*; C71*; C72*; C73*; C74*; C75*; C76*; C81*; C82*; C83*; C84*; C85*; C88*; C90*; C91*; C92*; C93*; C94*; C95*; C96*; C97* | |
| Carcinoma with metastasis | C77*; C78*; C79*; C80* | |
| Cerebrovascular disease | I6*; G45*; G46* | |
| Chronic ischemic heart disease | I20*; I25.0*; I25.1*; I25.5*; I25.6*; I25.8*; I25.9*; I24.9*; I24.8*; I250*; I251*; I255*; I256*; I259*; I249*; I248* | |
| Cognitive troubles/dementia | F00*; F01*; F02*; F03*; F05*; G30*; G31.1* | |
| Connective tissue disease | M05*; M06*; M315*; M32*; M33*; M34*; M351*; M353*; M360* | |
| Diabetes with organ damage | E10.2*; E10.3*; E10.4*; E10.5*; E10.7*; E11.2*; E11.3*; E11.4*; E11.5*; E11.7*; E12.2*;  E12.3*; E12.4*; E12.5*; E12.7*; E13.2*; E13.3*; E13.4*; E13.5*; E13.7*; E14.2*; E14.3*;  E14.4*; E14.5*; E14.7* | |
| Gastrointestinal ulcer | K25*; K26*; K27*; K28* | |
| Heart failure | I50*; I09.9*; I11.0*; I13.0*; I13.2*; I25.5*; I42.0*; I42.5*; I42.6*; I42.7*; I42.8*; I42.9*; I43*; P29.0* | |
| Hepatic cirrhosis | K70*; K71.7*; K74.3*; K74.4*; K74.5*; K74.6*; K717*; K743*; K744*; K745*; K746* | |
| Hypertension | I10*; I11*; I15*; I12*; I13* | |
| Infectious disease (except pneumonia and sepsis) | A*; B*; R65.0*; R65.1*; I33*; I40.0*; I41*; I43.0*; I52.1*; I68.1*; I98.1*; J00*; J2*; J44.0*; K67.8*; K77.0*; L0*; M00*; M01*; M46.3*; M46.4*; M49.3*; M60.0*; M63.1*; M63.2*; M65.1*; M71.1*; M90.1*; M90.2*; N39.0*; N43.1*; N77.0*; N77.1*; N98.0*; T79.3*; T80.2*; T81.4*; T82.6*; T82.7*; T83.5*; T83.6*; T84.5*; T84.6*; T84.7*; T85.7*; T87.4*; T88.0*; T89.0*  EXCEPT: B00.1; B00.9; B02.9, B18*; B23.8; B24; B35.1; B35.8; B36.0; B36.9; B37.0; B37.2; B37.3; B37.9; B90.9; B91; B95.2; B95.48  B95.6; B95.7; B95.8; B95.90; B95.91; B96.2; B96.3;B96.5; B96.6; B96.8; B96.81; B96.88; B97.8; B98.0;B99 ; A40*; A41*; B37.7*; A22.7*; A26.7*; A32.7*; A42.7* | |
| Intoxication or adverse drug reactions | T36*; T37*; T38*; T39*; T4*; T50*; T88*; Y5*  EXCEPT: T40.0; T40.1; T40.2; T40.5; T40.6; T40.7; T40.8; T40.9; | |
| Mental and behavioral disorders due to alcohol | F10*; G31.2*; G62.1*; G72.1*; I42.6*; K29.2*; K70*; K85.2*; K86.0*; Z50.2*; E24.4*; G312*; G621*; G721*; I426*; K292*; K852*; K860*; Z502*; E244* | |
| Paraplegia/hemiplegia | G041*; G114*; G801*; G802*; G81*; G82*; G830*; G831*; G832*; G833*; G834*; G839* | |
| **Variables of interest** | **Definition** | |
| Peripheral vascular disease | I70*; I71*; I731*; I738*; I739*; I771*; I790*; I792*; K551*; K558*; K559*; Z958*; Z959* | |
| Pneumonia | J13*; J14*; J15*; J16*; J17*; J18* | |
| Renal failure | I12.0*; I13.1*; N03.2*; N03.3*; N03.4*; N03.5*; N03.6*; N03.7*; N05.2*; N05.3*; N05.4*; N05.5*; N05.6*; N05.7*; N18*; N19*; N25.0*; Z49.0*; Z49.1*; Z49.2*; Z94.0*; Z99.2* | |
| Sepsis and septic choc | A40*; A41*; B37.7*; R57.2*; A22.7*; A26.7*; A32.7*; A42.7*; O85*; B377*; R572*; A227*; A267*; A327*; A427* |  |

Adapted from *Canadian Institute for Health Information, Hospital Standardized Mortality Ratio: Technical Notes, 2011.*

*S Table 1*: Variable of interest’s definition
