# Supplementary material for: Development of a predictive score for potentially avoidable hospital readmissions for general internal medicine patients
Source: PLoS One. 2019 Jul 15;14(7):e0219348. doi: 10.1371/journal.pone.0219348 (PMC6629067; doi:10.1371/journal.pone.0219348)
Supplement: S2 Table — AIDS: acquired immune deficiency syndrome; COPD: chronic obstructive pulmonary disease; ACE inhibitors: angiotensin-converting-enzyme inhibitors; NSAID: nonsteroidal anti-inflammatory drugs; ASAT: aspartate aminotransferase; ALAT: alanine aminotransferase; GGT: gamma-glutamyl transferase; AP: alkaline phosphatase. In bold: significant result (p < 0.05). S2 Table: Multivariate analysis with all variables of interest. (DOCX) [file pone.0219348.s002.docx]

**S Table 2: Multivariate analysis with all variables of interest**

| **Variables of interest** | **Beta** | ***P*-value** | **Odds ratio** | **95% CI** |
| --- | --- | --- | --- | --- |
| Intercept | -3.397 | < .0001 | - | - |
| Age 65–75 years | 0.195 | .126 | 1.22 | (0.95–1.56) |
| Age > 75 years | -0.085 | .502 | 0.92 | (0.72–1.18) |
| Male sex | -0.037 | .697 | 0.96 | (0.8–1.16) |
| **Length of stay > 4 days** | **0.269** | **.025** | **1.31** | **(1.04–1.66)** |
| **Admission in previous 6 months** | **0.832** | **.0001** | **2.30** | **(1.91–2.77)** |
| **Acute myocardial infarction** | **-0.452** | **.016** | **0.64** | **(0.44–0.91)** |
| Acute respiratory disease | 0.045 | .705 | 1.05 | (0.83–1.31) |
| AIDS | 0.267 | .724 | 1.31 | (0.20–4.63) |
| **Anemia** | **0.226** | **.024** | **1.25** | **(1.03–1.53)** |
| **Arrhythmia** | **0.262** | **.032** | **1.30** | **(1.02–1.65)** |
| COPD/asthma | 0.245 | .073 | 1.27 | (0.97–1.66) |
| **Cancer** | **0.346** | **.023** | **1.41** | **(1.04–1.89)** |
| **Carcinoma with metastasis** | **0.685** | **.001** | **1.98** | **(1.32–2.96)** |
| Cerebrovascular disease | -0.022 | .937 | 0.98 | (0.55–1.63) |
| **Chronic ischemic heart disease** | **0.53** | **.001** | **1.70** | **(1.24–2.31)** |
| Cognitive troubles/dementia | -0.211 | .494 | 0.81 | (0.42–1.43) |
| Connective tissue disease | 0.429 | .307 | 1.54 | (0.62–3.27) |
| **Diabetes with organ damage** | **0.81** | **.003** | **2.25** | **(1.30–3.81)** |
| Gastrointestinal ulcer | 0.304 | .374 | 1.35 | (0.66–2.54) |
| **Heart failure** | **0.272** | **.041** | **1.31** | **(1.01–1.70)** |
| Hepatic cirrhosis | 0.278 | .272 | 1.32 | (0.80–2.15) |
| **Hypertension** | **0.29** | **.013** | **1.34** | **(1.06–1.68)** |
| Infectious disease (except pneumonia and sepsis) | -0.192 | .113 | 0.83 | (0.65–1.04) |
| Intoxication or adverse drug reactions | 0.163 | .199 | 1.18 | (0.91–1.50) |
| Mental and behavioral disorders due to alcohol | -0.013 | .946 | 0.99 | (0.66–1.44) |
| Paraplegia/hemiplegia | 0.433 | .312 | 1.54 | (0.61–3.36) |
| Peripheric vascular disease | 0.317 | .187 | 1.37 | (0.84–2.16) |
| Pneumonia | 0.198 | .118 | 1.22 | (0.95–1.56) |
| Renal failure | 0.107 | .345 | 1.11 | (0.89–1.39) |
| Sepsis | -0.3 | .123 | 0.74 | (0.50–1.07) |
| Number of medications: 6–10 | -0.107 | .432 | 0.90 | (0.69–1.18) |
| Number of medications > 10 | -0.027 | .858 | 0.97 | (0.73–1.31) |
| ACE inhi./angiotensin II antag. | -0.076 | .493 | 0.93 | (0.75–1.15) |
| Antiplatelet drugs | -0.118 | .301 | 0.89 | (0.71–1.11) |
| Anticoagulants | -0.083 | .533 | 0.92 | (0.71–1.19) |
| Antipsychotics | -0.091 | .624 | 0.91 | (0.62–1.30) |
| Benzodiazepines | -0.173 | .075 | 0.84 | (0.70–1.02) |
| Beta blockers | 0.014 | .903 | 1.01 | (0.81–1.27) |
| Calcium-channel blockers | 0.034 | .773 | 1.04 | (0.82–1.30) |
| Digoxin | 0.227 | .358 | 1.26 | (0.76–2.00) |
| Diuretics | 0.121 | .31 | 1.13 | (0.89–1.42) |
| Hypoglycemic drugs (insulin/sulfonylurea/glinide) | 0.121 | .445 | 1.13 | (0.83–1.54) |

| **Variables of interest** | **Beta** | ***P*-value** | **Odds ratio** | **95% CI** |
| --- | --- | --- | --- | --- |
| Non-secretagogue antidiabetics | -0.136 | .446 | 0.87 | (0.61–1.23) |
| NSAIDs | 0.091 | .622 | 1.10 | (0.75–1.55) |
| **Opioids** | **0.281** | **.008** | **1.33** | **(1.07–1.63)** |
| Systemic anti-infectious drug | 0.004 | .972 | 1.00 | (0.81–1.25) |
| **Hyperkalemia (K > 5.5 mmol/L)** | **0.303** | **.031** | **1.35** | **(1.02–1.77)** |
| Hypokalemia (K < 3.5 mmol/L) | -0.124 | .218 | 0.88 | (0.72–1.07) |
| Hypernatremia (Na > 145 mmol/L) | -0.024 | .91 | 0.98 | (0.63–1.45) |
| Hyponatremia (Na < 135 mmol/L) | 0.045 | .654 | 1.05 | (0.86–1.27) |
| Liver dysfunction (ASAT/ALAT > 175; or total bilirubin > 40; or AP > 360; or GGT > 90). | -0.003 | .977 | 0.99 | (0.79–1.25) |

AIDS: acquired immune deficiency syndrome; COPD: chronic obstructive pulmonary disease; ACE inhibitors: angiotensin-converting-enzyme inhibitors; NSAID: nonsteroidal anti-inflammatory drugs; ASAT: aspartate aminotransferase; ALAT: alanine aminotransferase; GGT: gamma-glutamyl transferase; AP: alkaline phosphatase.

In bold: significant result (*p* < 0.05).

*S Table 2*: Multivariate analysis with all variables of interest
