# Supplementary material for: Development of a predictive score for potentially avoidable hospital readmissions for general internal medicine patients
Source: PLoS One. 2019 Jul 15;14(7):e0219348. doi: 10.1371/journal.pone.0219348 (PMC6629067; doi:10.1371/journal.pone.0219348)
Supplement: S3 Table — Calculator of predicted potentially avoidable readmission risk (+ Excel spreadsheet). (DOCX) [file pone.0219348.s003.docx]

**S Table 3: Calculator of predicted potentially avoidable readmission risk**

| **Variables** | **Yes = 1, No = 0** | **Points** | **Total** |  | **-3,46** |
| --- | --- | --- | --- | --- | --- |
| Previous admission within 6 months | 1 | 8 | 8 |  | 0,84 |
| Length of hospital stay > 4 days | 0 | 3 | 0 |  | 0 |
| Anemia | 0 | 2 | 0 |  | 0 |
| Heart failure | 1 | 4 | 4 |  | 0,37 |
| Hypertension | 0 | 3 | 0 |  | 0 |
| Acute myocardial infarction | 0 | -4 | 0 |  | 0 |
| Chronic ischemic heart disease | 1 | 5 | 5 |  | 0,49 |
| Diabetes with organ damage | 0 | 9 | 0 |  | 0 |
| Cancer | 0 | 4 | 0 |  | 0 |
| Metastatic carcinoma | 1 | 6 | 6 |  | 0,64 |
| Opioids | 1 | 3 | 3 |  | 0,27 |
| Hyperkalemia (> 5.5mmol/L) | 0 | 4 | 0 |  | 0 |
|  |  |  |  |  |  |
| **Total score** |  |  | **26** |  | -0,85 |
| **Predicted potentially avoidable readmission risk** |  |  |  |  | **30%** |

*S Table 3:* Calculator of predicted potentially avoidable readmission risk ( + *Excel* spreadsheet)
