# Supplementary material for: Development of a predictive score for potentially avoidable hospital readmissions for general internal medicine patients
Source: PLoS One. 2019 Jul 15;14(7):e0219348. doi: 10.1371/journal.pone.0219348 (PMC6629067; doi:10.1371/journal.pone.0219348)
Supplement: S4 Table — AIDS: acquired immune deficiency syndrome; COPD: chronic obstructive pulmonary disease; ACE inhibitors: angiotensin-converting-enzyme inhibitors; NSAID: nonsteroidal anti-inflammatory drugs; ASAT: aspartate aminotransferase; ALAT: alanine aminotransferase; GGT: gamma-glutamyl transferase; AP: alkaline phosphatase; PAR: potentially avoidable readmission. S4 Table: Baseline characteristics of the validation cohort. (DOCX) [file pone.0219348.s004.docx]

**S Table 4: Validation cohort’s baseline characteristics**

| **Baseline Characteristics** | **Entire Validation cohort (n = 3057)** | | **Non-PAR patients**  **(n = 2838)** | | **PAR patients**  **(n = 219)** | |
| --- | --- | --- | --- | --- | --- | --- |
|  | **No.** | % | **No.** | % | **No.** | % |
| University Hospitals of Geneva | 2133 | (69.8) | 1977 | (69.7) | 156 | (71.2) |
| Groupement Hospitalier de l’Ouest Lémanique | 924 | (30.2) | 861 | (30.3) | 63 | (28.8) |
| **Age** | | | | | | |
| ≤ 65 years | 1291 | (42.2) | 1221 | (43.0) | 70 | (32.0) |
| 66–75 years | 652 | (21.3) | 591 | (20.8) | 61 | (27.9) |
| ≥ 76 years | 1114 | (36.4) | 1026 | (36.2) | 88 | (40.2) |
| **Male, sex** | 1721 | (56.3) | 1594 | (56.2) | 127 | (58.0) |
| **Charlson Comorbidity Index** | | | | | | |
| ≤ 1 | 1677 | (54.9) | 1591 | (56.1) | 86 | (39.3) |
| 2–4 | 1052 | (34.4) | 963 | (33.9) | 89 | (40.6) |
| > 4 | 328 | (10.7) | 284 | (10.0) | 44 | (20.1) |
| **Length of hospital stay** | | | | | | |
| ≤ 4 days | 1095 | (35.8) | 1028 | (36.2) | 67 | (30.6) |
| > 4 days | 1962 | (64.2) | 1810 | (63. 8) | 152 | (69.4) |
| **Type of admission** | | | | | | |
| Unplanned, emergency | 2549 | (83,4) | 2399 | (84.5) | 150 | (68.5) |
| Planned | 316 | (10,3) | 298 | (10.5) | 18 | (8.2) |
| Transfer/others | 192 | (6,3) | 141 | (5.0) | 51 | (23.3) |
| **Admission in previous 6 months** | 864 | (28.3) | 760 | (26.8) | 104 | (47.5) |
| **Comorbidity:** | | | | | | |
| Acute myocardial infarction | 492 | (16.1 | 455 | (16.0) | 37 | (16.9) |
| Acute respiratory disease | 566 | (18.5) | 510 | (18.0) | 56 | (25.6) |
| AIDS | 7 | (0.2) | 6 | (0.2) | 1 | (0.5) |
| Anemia | 858 | (28.1) | 766 | (27.0) | 92 | (42.0) |
| Arrhythmia | 587 | (19.2) | 534 | (18.8) | 53 | (24.2) |
| COPD/asthma | 403 | (13.2) | 362 | (12.8) | 41 | (18.7) |
| Cancer | 343 | (11.2) | 290 | (10.2) | 53 | (24.2) |
| Carcinoma with metastasis | 127 | (4.1) | 103 | (3.6) | 24 | (11.0) |
| Cerebrovascular disease | 124 | (4.1) | 114 | (4.0) | 10 | (4.6) |
| Chronic ischemic heart disease | 262 | (8.6) | 237 | (8.3) | 25 | (11.4) |
| Cognitive troubles/dementia | 132 | (4.3) | 124 | (4.4) | 8 | (3.6) |
| Connective tissue disease | 34 | (1.1) | 34 | (1.2) | 0 | (0.0) |
| Diabetes with organ damage | 57 | (1.9) | 55 | (1.9) | 2 | (0.9) |
| Gastrointestinal ulcer | 24 | (0.8) | 22 | (0.8) | 2 | (0.9) |
| Heart failure | 526 | (17.2) | 472 | (16.6) | 54 | (24.67) |
| Hepatic cirrhosis | 103 | (3.4) | 97 | (3.4) | 6 | (2.7) |
| Hypertension | 1029 | (33.7) | 944 | (33.3) | 85 | (38.8) |
| Intoxication or adverse drug reactions | 376 | (12.3) | 342 | (12.1) | 34 | (15.5) |
| Infectious disease (except pneumonia and sepsis) | 741 | (24.2) | 691 | (24.3) | 50 | (22.8) |
| Mental and behavioral disorders due to alcohol | 266 | (8.7) | 252 | (8.9) | 14 | (6.4) |
| Paraplegia/hemiplegia | 48 | (1.6) | 43 | (1.5) | 5 | (2.3) |
| Peripheral vascular disease | 72 | (2.4) | 62 | (2.2) | 10 | (4.6) |
| Pneumonia | 485 | (15.9) | 451 | (15.9) | 34 | (15.5) |
| Renal failure | 691 | (22.6) | 634 | (22.3) | 57 | (26.0) |
| Sepsis | 243 | (7.9) | 222 | (7.8) | 21 | (9.6) |

| **Baseline Characteristics** | **Entire Validation cohort (n = 3,057)** | | | | **Non-PAR patients**  **(n = 2838)** | | | | **PAR patients**  **(n = 219)** | |
| --- | --- | --- | --- | --- | --- | --- | --- | --- | --- | --- |
|  | N | | % | | N | % | | | N | % |
| **Number of medications** | | | | | | | | | | |
| ≤ 5 | 1964 | | (64.3) | | 1834 | (64.6) | | | 130 | (59.3) |
| 6–10 | 374 | | (12.2) | | 339 | (12.0) | | | 35 | (16.0) |
| > 10 | 719 | | (23.5) | | 665 | (23.4) | | | 54 | (24.7) |
| **Main drug prescribed** | | | | | | | | | | |
| ACE inh./angiotensin II antag. | | 953 | | (31.2) | 883 | | (31.1) | 70 | | (32.0) |
| Antiplatelet drugs | | 884 | | (28.9) | 816 | | (28.7) | 68 | | (31.0) |
| Anticoagulants | | 456 | | (14.9) | 422 | | (14.9) | 34 | | (15.5) |
| Antipsychotics | | 202 | | (6.6) | 190 | | (6.7) | 12 | | (5.5) |
| Benzodiazepines | | 1128 | | (36.9) | 1052 | | (37.1) | 76 | | (34.7) |
| Beta blockers | | 868 | | (28.4) | 800 | | (28.2) | 68 | | (31.0) |
| Calcium channel blockers | | 464 | | (15.2) | 423 | | (14.9) | 41 | | (18.7) |
| Digoxin | | 53 | | (1.7) | 47 | | (1.7) | 6 | | (2.7) |
| Diuretics | | 881 | | (28.8) | 802 | | (28.3) | 79 | | (36.1) |
| Hypoglycemic drugs (insulin/sulfonylurea/glinides) | | 344 | | (11.2) | 321 | | (11.3) | 23 | | (10.5) |
| Non-secretagogue antidiabetics | | 260 | | (8.5) | 247 | | (8.7) | 13 | | (5.9) |
| NSAID | | 267 | | (8.7) | 251 | | (8.8) | 16 | | (7.3) |
| Opioids | | 713 | | (23.3) | 646 | | (22.8) | 67 | | (30.6) |
| Systemic anti-infectious drugs | | 1132 | | (37.0) | 1051 | | (37.0) | 81 | | (37.0) |
| **Laboratory analysis** | | | | | | | | | | |
| Hyperkalemia (K > 5.5 mmol/L) | | 248 | | (8.1) | 217 | | (7.6) | 31 | | (14.2) |
| Hypokalemia (K < 3.5 mmol/L) | | 1208 | | (39.5) | 1115 | | (39.3) | 93 | | (42.5) |
| Hypernatremia (Na > 145 mmol/L) | | 139 | | (4.5) | 123 | | (4.3) | 16 | | (7.3) |
| Hyponatremia (Na < 135 mmol/L) | | 949 | | (31.0) | 851 | | (30.0) | 98 | | (44.7) |
| Liver dysfunction (ASAT/ALAT > 175; or total bilirubin > 40; or AP > 360; or GGT > 90) | | 809 | | (26.5) | 739 | | (26.0) | 70 | | (32.0) |

AIDS: acquired immune deficiency syndrome; COPD: chronic obstructive pulmonary disease; ACE inhibitors: angiotensin-converting-enzyme inhibitors; NSAID: nonsteroidal anti-inflammatory drugs; ASAT: aspartate aminotransferase; ALAT: alanine aminotransferase; GGT: gamma-glutamyl transferase; AP: alkaline phosphatase

PAR: potentially avoidable readmission

*S Table 4*: Baseline characteristics of the validation cohort
