# Supplementary material for: Development of a predictive score for potentially avoidable hospital readmissions for general internal medicine patients
Source: PLoS One. 2019 Jul 15;14(7):e0219348. doi: 10.1371/journal.pone.0219348 (PMC6629067; doi:10.1371/journal.pone.0219348)
Supplement: S5 Table — A) In derivation cohort (n = 7,317) B) In validation cohort (n = 3,057). S5 Table: Predicted number of potentially avoidable hospital readmissions by decile, in both derivation and validation cohorts. (DOCX) [file pone.0219348.s005.docx]

**S Table 5: Predicted number of potentially avoidable hospital readmissions by decile, in both derivation and validation cohorts**

1. In derivation cohort (n = 7,317)

| **Decile** | **Score** | **Mean score** | **Patients repartition**  **n (%)** | | **No. of potentially avoidable readmissions** | **Mean risk observed** | **Mean risk predicted** |
| --- | --- | --- | --- | --- | --- | --- | --- |
| 1 | -4 to -1 | -1.61 | 183 | (2.5%) | 5 | 2.7 | 2.6 |
| 2 | 0–2 | 0.42 | 999 | (13.7%) | 26 | 2.6 | 3.2 |
| 3 | 3–4 | 3.10 | 1,466 | (20.0%) | 45 | 3.1 | 4.0 |
| 4 | 5–6 | 5.58 | 980 | (13.4%) | 52 | 5.3 | 5.1 |
| 5 | 7–8 | 7.57 | 719 | (9.8%) | 59 | 8.2 | 6.2 |
| 6 | 9–10 | 9.55 | 566 | (7.7%) | 47 | 8.3 | 7.4 |
| 7 | 11–13 | 11.75 | 907 | (12.4%) | 81 | 8.9 | 9.2 |
| 8 | 14–16 | 14.90 | 659 | (9.0%) | 91 | 13.8 | 12.1 |
| 9 | > 16 | 20.57 | 838 | (11.5%) | 156 | 18.6 | 19.9 |

1. In validation cohort (n = 3,057)

| **Decile** | **Score** | **Mean score** | **Patients repartition n (%)** | | **No. of potentially avoidable readmissions** | **Mean risk observed** | **Mean risk predicted** |
| --- | --- | --- | --- | --- | --- | --- | --- |
| 1 | -4 to -1 | -1.61 | 64 | (2.1%) | 0 | 0 | 2.6 |
| 2 | 0–2 | 0.41 | 466 | (15.2%) | 14 | 3.0 | 3.2 |
| 3 | 3–4 | 3.10 | 557 | (18.2%) | 13 | 2.3 | 4.0 |
| 4 | 5–6 | 5.61 | 410 | (13.4%) | 20 | 4.9 | 5.1 |
| 5 | 7–8 | 7.54 | 304 | (9.9%) | 21 | 6.9 | 6.2 |
| 6 | 9–10 | 9.56 | 228 | (7.5%) | 18 | 7.8 | 7.3 |
| 7 | 11–13 | 11.83 | 374 | (12.2%) | 47 | 12.6 | 9.3 |
| 8 | 14–16 | 14.90 | 281 | (9.2%) | 35 | 12.4 | 12.1 |
| 9 | > 16 | 20.42 | 373 | (12.2%) | 51 | 13.7 | 19.7 |

*S Table 5:* Predicted number of potentially avoidable hospital readmissions by decile, in both derivation and validation cohorts
